# Synergistic effect of the anti-PD-1 antibody with blood stable and reduction sensitive curcumin micelles on colon cancer

**DOI:** 10.1080/10717544.2021.1921077

**Published:** 2021-05-11

**Authors:** Feirong Gong, Jian-Chao Ma, Jianguo Jia, Fa-Zhan Li, Jiao-Lan Wu, Shanfeng Wang, Xin Teng, Zhong-Kai Cui

**Affiliations:** a Key Laboratory for Ultrafine Materials of Ministry of Education, School of Materials Science and Engineering, East China University of Science and Technology, Shanghai, China; b Department of Cell Biology, School of Basic Medical Sciences, Southern Medical University, Guangzhou, China; c Guangdong Provincial Key Laboratory of Bone and Joint Degeneration Diseases, The Third Affiliated Hospital, Southern Medical University, Guangzhou, China; d Department of Cardiology, Shanghai Institute of Cardiovascular Disease, Zhongshan Hospital, Fudan University, Shanghai, China; e School of Materials Science and Engineering, Sun Yat-sen University, Guangzhou, China

**Keywords:** Telodendrimer micelles, glutathione-triggered release, bioavailability, synergistic effect, cancer immunotherapy

## Abstract

Curcumin (1,7-bis(4-hydroxy-3-methoxyphenyl)-1,6-heptadiene-3,5-dione) is a potent anticancer drug with versatile biological activities, while the clinical translation of curcumin is severely limited due to its hydrophobicity, rapid elimination, and metabolism in the blood circulation. Herein, we aim to unravel the potential of curcumin as a synergistic agent with immunotherapy in the treatment of cancers. In an effort to minimize premature release and improve the systemic bioavailability, a superior blood stable and reduction sensitive curcumin micellar formulation, of which the release can be triggered by cancer cells, is rationally designed. We have synthesized a telodendrimer (mPEG-PLA-(LA)_4_) capable of forming reversible disulfide crosslinked micelles (DCMs). The curcumin loaded DCMs (Cur/DCMs) are spherical with a uniform size of 24.6 nm. The *in vitro* release profile demonstrates that curcumin releases significantly slower from DCMs than that from non-crosslinked micelles (NCMs), while the release can be accelerated with the increasing concentration of reducing agent glutathione (GSH). Intravenous administration of Cur/DCMs stably retains curcumin in the bloodstream and efficiently improves the systemic bioavailability. Furthermore, Cur/DCMs exhibit synergistic anticancer efficacy when combined with the anti-PD-1 antibody in an MC-38 colon cancer xenograft model. Our results potentiate the integration of blood stable curcumin nanoformulation and immunotherapy for cancer treatment.

## 1. Introduction

Curcumin, a bright yellow lipophilic polyphenol derived from the *Curcuma longa* plants, is well known for its antitumor potential, as it is nontoxic and possesses versatile biological activities, including anti-oxidant, anti-inflammatory, anti-proliferative, and anti-angiogenesis (Dhillon et al., 2008; Aggarwal & Harikumar, 2009; Basnet & Skalko-Basnet, 2011; Kanai et al., 2011). In addition, curcumin has exhibited its potential in overcoming multidrug resistance and a synergistic effect with other anticancer agents for reducing toxicity and improving efficacy in some preclinical models (Verma et al., 1997; Khafif et al., 1998; Tang et al., 2005; Weir et al., 2007; Ganta & Amiji, 2009; Hu & Zhang, 2012). Interest in the therapeutic application of curcumin in cancer therapy has led to extensive investigations. A few formulations of redox-responsive curcumin nanoparticles were prepared for tumor treatment (Cao et al., 2015; Meng et al., 2018; Wang et al., 2018). Unfortunately, the clinical translation of curcumin as an anticancer agent has been severely limited. Promising therapeutic effects of curcumin have been observed *in vitro*; however, its efficacy *in vivo* is usually inadequate and does not reflect the *in vitro* results. In recent years, the putative anticancer properties of curcumin have resulted in several clinical trials against various tumors and in some cases positive trends that warrant further study were documented. However, limited success has been achieved in humans (Nelson et al., 2017), mainly because of its low bioavailability and confusing indications. First, curcumin is water insoluble and undergoes rapid transformation at physiological conditions, resulting in poor stability, rapid elimination and metabolism, limited cellular uptake, and minimal bioavailability (Sharma et al., 2005; Anand et al., 2007). On the other hand, although curcumin was reported to suppress various types of cancers including pancreas, prostate, leukemia, bladder, etc., no significant benefits have been confirmed by double blinded, placebo controlled clinical trials (Nelson et al., 2017). The indication of curcumin to play a role in cancer therapy is still ambiguous.

Over the past few years, the field of cancer immunotherapy has entered a new and exciting era, spurred by the extended understanding on the complex relationship between the tumor and the immune system (Robert et al., 2013). Human carcinoma cells can activate intrinsic programmed cell death in lymphocytes interacting with the tumor (Philips & Atkins, 2015), which avoids immune recognition as well as elimination and promotes tumor growth and metastasis. Modern designed immunotherapeutic agents intend to stimulate immune responses, such that antibodies targeting either programmed-death-protein-1 receptor (PD-1) or its ligand (PD-L1) have stimulated significant antitumor activity with considerably less toxicity (Philips & Atkins, 2015; Alsaab et al., 2017). A major advantage of these agents is the long-lasting clinical benefit, while the setback is that so far only a prospectively unidentified proportion of patients (approximately 25%) with solid tumors experiences clinical benefits. Unfortunately, some types of tumors, such as bladder and head and neck cancer, are hardly sensitive to immunotherapy.

Curcumin inhibits myeloid-derived suppressor cells (MDSCs) in the spleen and tumor tissues, which strongly impair the T-cell function and contribute to immune suppression (Tu et al., 2012). Previous reports also have shown a strong immunomodulatory capability of curcumin by improving the status of T lymphocytes in peripheral blood restricts tumor-induced loss of thymic T cells in tumor-bearing mice (Bhattacharyya et al., 2007; Chang et al., 2012; Zhao et al., 2012). In addition, curcumin improves immunotherapeutic activities of vaccine to late stage tumors through breaking down the innate and adaptive system barriers and reversing the immunosuppressive tumor microenvironment in an advanced melanoma model (Lu et al., 2016).

**Figure 1.**
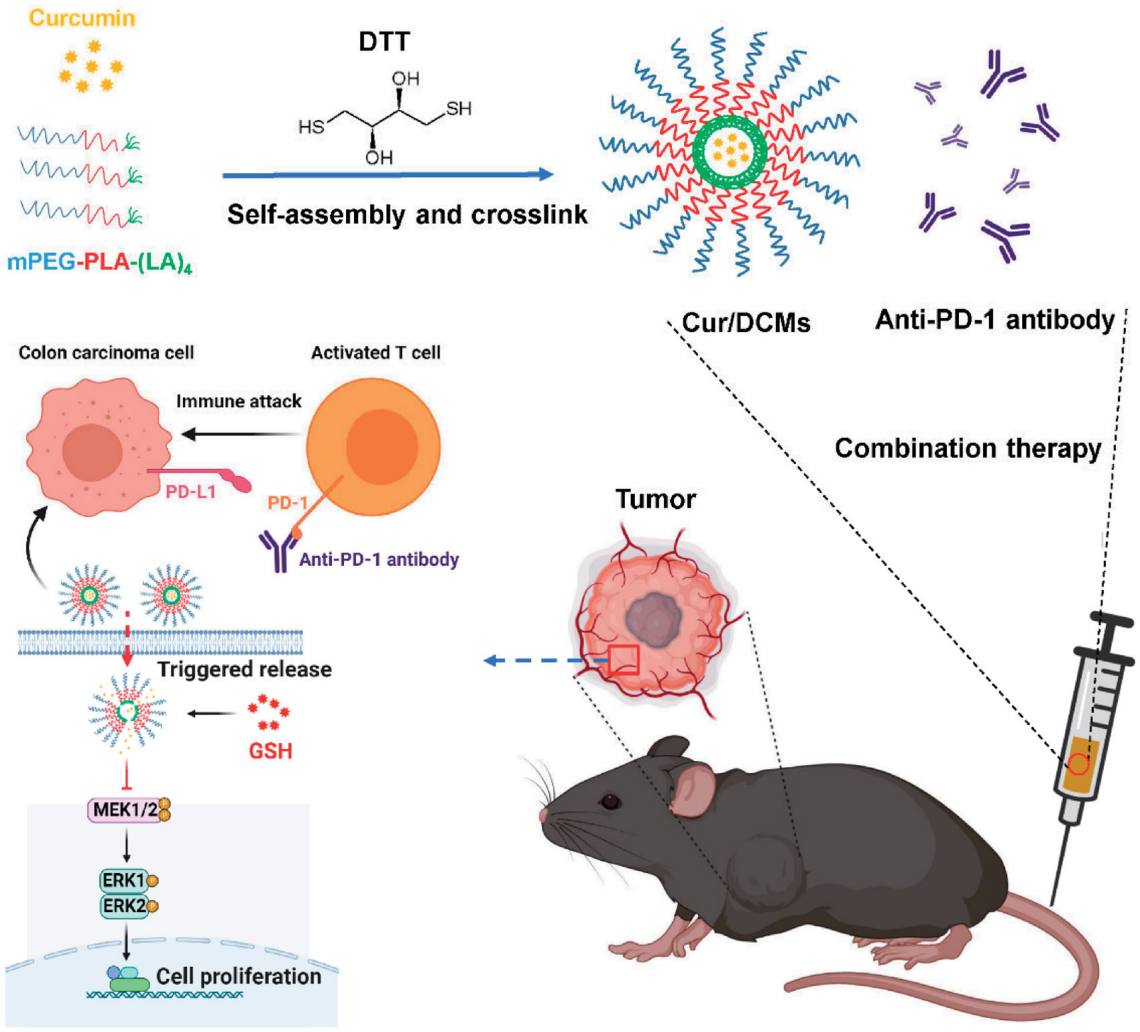
The schematic illustration of the formation process of the Cur/DCMs and the intracellular mechanism of the combination therapy.

We hypothesize that curcumin may synergize the therapeutic intervention of immunotherapeutic agents through various mechanisms. In the present study, a superior blood stable and reduction sensitive curcumin micellar formulation was designed and prepared, in order to increase the bioavailability of curcumin as a means to enhance its biological activities. Curcumin was encapsulated in the disulfide crosslinked core of the micelles and its stability both *in vitro* and *in vivo* was assessed. Disulfide crosslinking was employed to confer the triggered release to the micelles. Subsequently, *in vivo* antitumor efficacy with the anti-PD-1 antibody was evaluated (Figure 1).

## 2. Materials and methods

### 2.1. Materials

Curcumin was supplied by Soochow Nanomedicine Company (Soochow, China) with a purity of >99.5%. Methoxy poly(ethylene glycol) (mPEG, *M*_n_ = 2000 g/mol, PDI = 1.03), d,l-lactide, stannous octoate (Sn(Oct)_2_), l-glutathione (GSH), glutathione monoethyl ester (GSH-OEt), and dithiothreitol (DTT) were purchased from Sigma-Aldrich (Milwaukee, WI). Acetonide-2,2-dimethylolpropanoic anhydride (Ac-DMPA, 1) was synthesized according to a previous report (Gillies & Frechet, 2002). Dowex H^+^ resin (200–400 mesh), 4-pyrrolidinopyridine (4-py), and pivaloyl chloride were purchased from Acros (Beijing, China). Triethylamine (TEA), ethyl acetate, ether, anhydrous dichloromethane and ethanol were purchased from Shanghai Titan Scientific Co., Ltd. (Shanghai, China). Anti-PD-1 antibody (PD-1) was purchased from Wuxi AppTec Co. Ltd. (Shanghai, China). All other chemicals were of analytical grade from Sinopharm Chemical Regent Co. Ltd. (Shanghai, China) and used without further purification.

### 2.2. Cells and animals

Normal human colon epithelial cell line (NCM460) was purchased from INCELL Corporation (San Antonio, TX) and cultured in M3 media (San Antonio, TX) supplemented with 10% fetal bovine serum (FBS), 1% antibiotics (100 U/mL penicillin and 100 µg/mL streptomycin) (Burlington, Canada) at 37 °C in a humidified 5% CO_2_ atmosphere. Murine colon adenocarcinoma cells (MC-38) were obtained from American Type Culture Collection (ATCC, Manassas, VA) and cultured in Roswell Park Memorial Institute (RPMI) 1640 medium (Invitrogen GmbH, Karlsruhe, Germany) containing 10% FBS and 1% antibiotics at 37 °C in a humidified 5% CO_2_ atmosphere.

Male and female evenly Sprague-Dawley rats (SD rats) with specific pathogen free (SPF) grade (250 ± 5 g) and C57BL/6 mice (21 ± 2 g, 4–6 weeks old) were purchased from Vital River (Beijing, China). All animals were housed in a room maintained at 23 ± 3 °C, 65–75% humidity, with a controlled 12 h light–dark cycle for 5–7 days before experiments. Water and commercial laboratory complete food for animals were available. All animal procedures were conducted following the protocol approved by the Institutional Animal Care and Use Committee of Mabspace Biosciences Co. (Soochow, China).

### 2.3. Characterization

The molecular weight and polydispersity of the synthesized polymers were determined by a Waters 1515 gel permeation chromatographic (GPC) instrument equipped with a differential refractive-index detector. The measurements were performed using tetrahydrofuran (THF) as the eluent at a flow rate of 1.0 mL/min at 30 °C and polystyrene standards for the calibration of the columns. ^1^H nuclear magnetic resonance (^1^H NMR) spectra were obtained in deuterated chloroform (CDCl_3_) for validating the chemical structure and calculating the functionality of terminal hydroxyl groups in mPEG-PLA-(OH)_4_ with lipoic acid using a Bruker NMR spectrometer (AVANCE III, 500 MHz, Billerica, MA) at 25 °C. The morphologies of the curcumin-loaded micelles before and after crosslinking were examined using a JEM-2100 transmission electron microscope (TEM, JEOL, Tokyo, Japan). The thermal properties of the polymers were characterized on a differential scanning calorimeter (DSC, DSC-SP, Rheometric Scientific, Piscataway, NJ) through a heating cycle from 20 to 100 °C under nitrogen atmosphere at 10 °C/min and the curves were recorded for the second run. The mean diameter and size distribution of the micelles were determined using dynamic light scattering (DLS, Zetasizer Nano-ZS, Malvern Instruments, Malvern, UK). The measurements were performed in triplicate. The curcumin concentrations in the micelle dispersions were quantified at 25 °C using Agilent 1260 high-performance liquid chromatography (HPLC) (Agilent Technologies, Santa Clara, CA). The wavelength of the detector was 425 nm. The eluent was a mixture of acetonitrile/water (75/25) and the micelle dispersion was diluted with acetonitrile and filtered using the polyvinylidene fluoride (PVDF) filter before it flowed through a SB-C18 chromatographic column at 1 mL/min. The encapsulation efficiency and loading capacity of the drug were calculated according to Equation (1) and (2), respectively. The weight of drug in micelles was derived from the curcumin concentration in the micelle dispersions, while the weight of the initial drug included free curcumin that was not encapsulated and later removed before the HPLC determination.

(1) $$\text{Encapsulation efficiency}=\frac{\text{weight of drug in micelles}}{\text{weight of the initial drug}}\times 100\%$$

(2) $$\text{Loading capacity}=\frac{\text{weight of drug in micelles}}{\text{weight of micelles and drug}}\times 100\%$$

### 2.4. Synthesis of mPEG-PLA-OH

Mono-hydroxyl-terminated mPEG-PLA (mPEG-PLA-OH) was synthesized by a ring-opening polymerization of d,l-lactide in the presence of Sn(OCt)_2_. Briefly, mPEG2000 (15 g, 7.5 mmol) was added in a Schlenk bottle and degassed at 130 °C under reduced pressure with magnetic stirring for 2 h to eliminate the residue water. d,l-Lactide (5 g, 34.7 mmol) and Sn(Oct)_2_ (5 mg, 12.3 nmol) in anhydrous dichloromethane were added into the bottle in a glove-box. Then, the bottle was purged with nitrogen and degassed at high vacuum for 2 h at room temperature to remove the solvent. Then, the bottle was sealed and maintained at 130 °C under stirring for 15 h. The synthesized mPEG-PLA-OH with a number molecular weight of 500–2000 was recovered by dissolving in dichloromethane followed by precipitation in cold ether. The resultant precipitates were filtered and dried under vacuum at room temperature for 24 h.

### 2.5. Synthesis of mPEG-PLA-Ac

mPEG-PLA-OH (15 g, 6 mmol), TEA (2.50 mL, 18.0 mmol), and 4-py (267 mg, 1.80 mmol) were dissolved in 150 mL of dichloromethane in an oven dried flask. Then, 5.85 g of Ac-DMPA (18.0 mmol) was added and the mixture was stirred at room temperature for 6 h. After the completion of the reaction, the solvent was evaporated and the residue was recrystallized from cold ethanol for three times. After filtration and drying in vacuum at room temperature for 24 h, acetonide-2,2-dimethylol propanoic acid terminated mPEG-PLA (mPEG-PLA-Ac) was obtained as a white solid.

### 2.6. Synthesis of mPEG-PLA-(OH)_2_

mPEG-PLA-Ac (15.0 g, 3.74 mmol) was dissolved in 50 mL of methanol and three tea spoons of Dowex H^+^ resin was added. The mixture was stirred at room temperature for 24 h. After filtration, the polymer solution was precipitated in cold ether and the resultant precipitates (mPEG-PLA-(OH)_2_) were collected after filtration and dried under vacuum at room temperature for 24 h.

### 2.7. Synthesis of mPEG-PLA-(Ac)_2_

mPEG-PLA-(OH)_2_ (10.0 g, 3.80 mmol), TEA (3.16 mL, 22.8 mmol), and 4-py (340 mg, 2.28 mmol) were dissolved in 100 mL of dichloromethane in an oven dried flask. Then, 7.42 g of Ac-DMPA (22.8 mmol) was added and the mixture was stirred at room temperature for 24 h. After the completion of the reaction, the solvent was evaporated and the residue was recrystallized from cold ethanol for three times. After filtration and drying in vacuum at room temperature for 24 h, mPEG-PLA-(Ac)_2_ was obtained as a white solid.

### 2.8. Synthesis of mPEG-PLA-(OH)_4_

Ten grams of mPEG-PLA-(Ac)_2_ (3.36 mmol) was dissolved in 100 mL of methanol and four tea spoons of Dowex H^+^ resin was added. The mixture was stirred at room temperature for 24 h. After filtration, the polymer solution was precipitated in cold ether and the resultant precipitates mPEG-PLA-(OH)_4_ were collected after filtration and drying under vacuum at room temperature for 24 h.

#### 2.9. Synthesis of mPEG-PLA-(LA)_4_

Lipoic acid (5.70 g, 27.6 mmol) and TEA (3.82 mL, 27.6 mmol) were dissolved in 50 mL of anhydrous ethyl acetate at −10 °C. Then, 3.5 mL of pivaloyl chloride (27.6 mmol) was slowly added. A white precipitate appeared immediately. The mixture was stirred at 0 °C for 2 h and then at room temperature for 1 h. The insoluble TEA–HCl was filtered off, the solvent was evaporated, and the residue was dried in vacuum for 1 h. The obtained viscous yellow oil was dissolved in 50 mL of anhydrous dichloromethane and cannulated into a chilled solution of mPEG-PLA-(OH)_4_ (6.7 g, 2.3 mmol), TEA (4.0 mL, 27.7 mmol), and 4-py (410 mg, 2.77 mmol) in 70 mL of dichloromethane. The solution was stirred at room temperature for 24 h. After the completion of the reaction, the solvent was evaporated and the residue was recrystallized from cold ethanol for three times. The precipitated mPEG-PLA-(LA)_4_ was collected by filtration and dried in vacuum at room temperature for 24 h.

### 2.10. Preparation and stability evaluation of Cur/DCMs

Curcumin-loaded noncrosslinked micelles (Cur/NCMs) were prepared by a solid dispersion and thin film hydration method. Typically, 20 mg of curcumin and 380 mg of mPEG-PLA-(LA)_4_ were dissolved in 5 mL ethyl acetate at 40 °C. The solvent was slowly evaporated under vacuum to form a thin film followed by hydration of the film with 5 mL of ultrapure water. The micelle dispersion was filtered using a PVDF filter (0.22 μm) to remove curcumin that was not encapsulated and such a formulation typically contained ∼5% curcumin and ∼95% mPEG-PLA-(LA)_4_.

Curcumin loaded DCMs (Cur/DCMs) were obtained by the ring-opening polymerization of disulfide-containing lipoyl units using DTT as the catalyst, as reported previously (Noda et al., 1990; Gong et al., 2017). Briefly, 1 mL of Tris–HCl buffer (50 mM, pH 8.5) was added to the above Cur/NCMs dispersion. After vacuum and purging nitrogen into the bottle, 13.2 mg DTT (85.6 µmol, 20 mol% relative to the lipoyl units) in 1 mL of water was added and the mixture was stirred at room temperature for 1 h, and then dialyzed against water for 6 h using a dialysis bag (MWCO 8000–14,000). The water was refreshed every one hour. Blank NCMs and DCMs were also prepared following the same protocol. After lyophilization, molecular weight of the micelles before and after crosslinking was characterized by GPC. The stability of Cur/NCMs and Cur/DCMs in saline, 50% ethanol and 50% sodium dodecyl sulfate (SDS) (2.5 mg/mL) was also monitored using DLS.

### 2.11. Triggered release of curcumin *in vitro*

In order to evaluate the crosslinking effect, *in vitro* release profiles of curcumin from DMSO (s-Cur), Cur/NCMs, and Cur/DCMs were studied by dynamic dialysis method with 20% SDS solution as release medium. Lyophilized micelles were reconstituted and diluted to 1 mg/mL of curcumin with PBS (0.1 M, pH 7.4) then placed 1 mL into dialysis tube (MWCO 8000–14,000 Da). The tubes were dialyzed against 50 mL release medium at 37 ± 0.5 °C at a stirring speed of 100 rpm with or without 10 mM GSH. At predetermined time intervals, 1 mL of release medium was withdrawn and replenished with an equal volume of the fresh medium. The amount of curcumin released was determined by HPLC analysis as mentioned above. Cumulative release of curcumin was then calculated. The *in vitro* release studies were carried out in triplicate.

### 2.12. *In vitro* cytotoxicity assay

The cytotoxicity of s-Cur, Cur/NCMs, and Cur/DCMs against MC-38 cells was determined by a CCK-8 assay. Briefly, MC-38 cells were cultured in RPMI1640 medium with 10% FBS, penicillin (100 U/mL), and streptomycin (100 µg/mL) for 24 h. Then, the cells were seeded in 96-well plates (Corning, NY) at 10,000 cells/well in 100 µL medium. After incubation for 24 h, the cells were exposed to s-Cur and equivalent amount of Cur/NCMs and Cur/DCMs to yield final curcumin concentrations from 0 to 65 µg/mL. For GSH triggered release experiments, adherent cells were incubated with 10 mM glutathione monoethyl ester (GSH-OEt) for 2 h before exposure to the curcumin formulations.

After incubation for 72 h, 10 μL CCK8 solution with 100 μL growth medium was added into each well and the cells were incubated for another 1 h. Then, the culture medium was added with 10 μL 1% SDS (dissolve 0.1 g SDS with PBS to obtain 10 mL solution) to stop the reaction, and reading on a Synergy HTX multi-mode reader (BioTek, Winooski, VT) at 450 nm. The cytotoxicity assay was performed in triplicate and the cell viability (%) was calculated with the following equation:

(3) $$\text{Cell viability}=\frac{A_{t}-A_{B}}{A_{c}-A_{B}}\times 100\%$$

where *A_t_* is the absorption value of samples, *A_c_* is the absorption value of the control group, and *A_B_* is the absorption value of the blank group. The viability of NCM460 and MC-38 cells incubated with various concentrations of blank NCMs and DCMs for 72 h was also evaluated.

### 2.13. Hemolysis assay

Human whole blood samples were collected from a volunteer in an ethylenediaminetetraacetic acid (EDTA) precoated tube. The authors assert that all procedures comply with the ethical standards of the relevant national and institutional committees on human experimentation and with the Helsinki Declaration of 1975, as revised in 2008. Written informed consent was obtained from the volunteer before blood draw in this study. Five milliliters whole blood was transferred into a tube with 10 mL calcium- and magnesium-free Dulbecco’s phosphate buffered saline (PBS, Grand Island, NY) and centrifuged at 500×*g* for 10 min to isolate RBCs. This purification step was repeated five times, and then the washed RBCs were diluted with PBS to 50 mL. To test the hemolytic activity of NCMs and DCMs, 0.2 mL of diluted RBC suspension (∼4.5 × 10^8^ cells/mL) was mixed with 0.8 mL of NCMs or DCMs suspension in PBS. The final concentration of NCMs and DCMs ranges from 0.001 to 1000 μg/mL. D.I. water (+RBCs) and PBS (+RBCs) were used as the positive control and negative control, respectively. All samples were placed on a rocking shaker in an incubator at 37 °C for 3 h. After incubation, the samples were centrifuged at 10,016×*g* for 3 min. The hemoglobin absorption in the supernatant was measured at 540 nm, with 655 nm as a reference, using a Synergy HTX multi-mode reader. Percent hemolysis was calculated with the following equation:

(4) $$\text{Percent hemolysis\%}=\frac{A_{s}-A_{nc}}{A_{pc}-A_{nc}}\times 100$$

where *A_s_*, *A_nc_*, and *A_pc_* are the absorption values of the samples, the negative control, and the positive control, respectively.

### 2.14. Western blot assay

MC38 cells were lysed using whole protein extraction kit (KeyGEN BioTECH, Nanjing, China, KGP250). Protein concentration was determined using bicinchoninic acid (BCA) protein assay (KeyGEN BioTECH, Nanjing, China, KGPBCA). Protein extracts were electrophoresed in 10% SDS-PAGE and transferred to a nitrocellulose filter (NC) membrane, blocked by 5% skim milk at room temperature for 1 h, which was prepared in Tris-buffered saline with tween 20 (TBST). Then, the membranes were immunoblotted by primary antibodies. For detection, HRP-conjugated secondary antibodies (Ray Antibody Biotech, Beijing, China, RM2001L) and chemiluminescent HRP substrate kit (EpiZyme, Shanghai, China, SQ2O2) were used. As the molecular weight of the target bands is similar, the western blot fast stripping buffer (EpiZyme, Shanghai, China, PS107) was used. The following primary antibodies were used: anti-p-MEK1/2 (Cell Signaling Technology (CST), 41G9, Boston, MA), anti-p-Erk1/2 (CST, 197G2). The blots were quantified using ImageJ (National Institutes of Health, Bethesda, MD) and normalized to the control group.

### 2.15. Pharmacokinetics

Nine SD rats were randomly divided into three groups (three rats for each group), receiving s-Cur, Cur/NCMs, and Cur/DCMs. Following the intravenous (i.v.) administration of the three curcumin formulations (20 mg/kg), 0.1 mL of the blood samples was collected from the tail vein at predetermined time intervals. The blood samples were centrifuged for 6 min at 5867×*g* to obtain the plasma. Then, the curcumin concentrations in the plasma were analyzed using a liquid chromatography mass spectrometer (LCMS-2020) equipped with a Shimadzu UV-visible spectrophotometer (Columbia, MD). The detection limitation of the equipment was 1 ng/mL. We employed the non-compartmental analysis in WinNonlin software V6.2.1 to calculate major pharmacokinetic parameters.

### 2.16. *In vivo* antitumor efficacy

The therapeutic efficacy of saline, Cur/DCMs, PD-1, and the combination therapy of Cur/DCMs and PD-1 was examined on C57BL/6 mice bearing MC-38 colon tumor. MC-38 cells were suspended in RPMI1640 culture medium containing 10% FBS. Suspension of 3 × 10^5^ cells in 100 μL medium was injected subcutaneously into animal armpits. Once the mass of the tumor in the xenografts reached ∼70 mm^3^, the mice were divided into four random groups (six animals per group) for receiving physiological saline (100 µL every day), Cur/DCMs (i.v., 40 mg/kg every day), PD-1 (i.v., 10 mg/kg every week), and combination therapy of Cur/DCMs (i.v., 40 mg/kg every day) and PD-1 (i.v., 10 mg/kg every week). After the initial treatment, the mice were continuously monitored for 21 days in terms of body weight and tumor dimensions (length and width). According to the animal welfare, once the tumor volume exceeds 2000 mm^3^, the mice were euthanized. Tumor volume was calculated according to the following equation:

(5) $$V=\frac{1}{2}\mathrm{length}\times {\mathrm{width}}^{2}.$$

### 2.17. Statistical analysis

All values presented in this work were the average of at least three independent experiments unless otherwise stated, and the error bars represent the standard deviations. The difference between any two treatment groups was determined using one-way ANOVA, followed by Tukey’s post hoc or nonparametric test (SPSS version 17.0, Chicago, IL). *p*< 0.05 indicated statistical significance.

## 3. Results and discussion

### 3.1. Synthesis and characterization of mPEG-PLA-(LA)_4_

**Figure 2.**
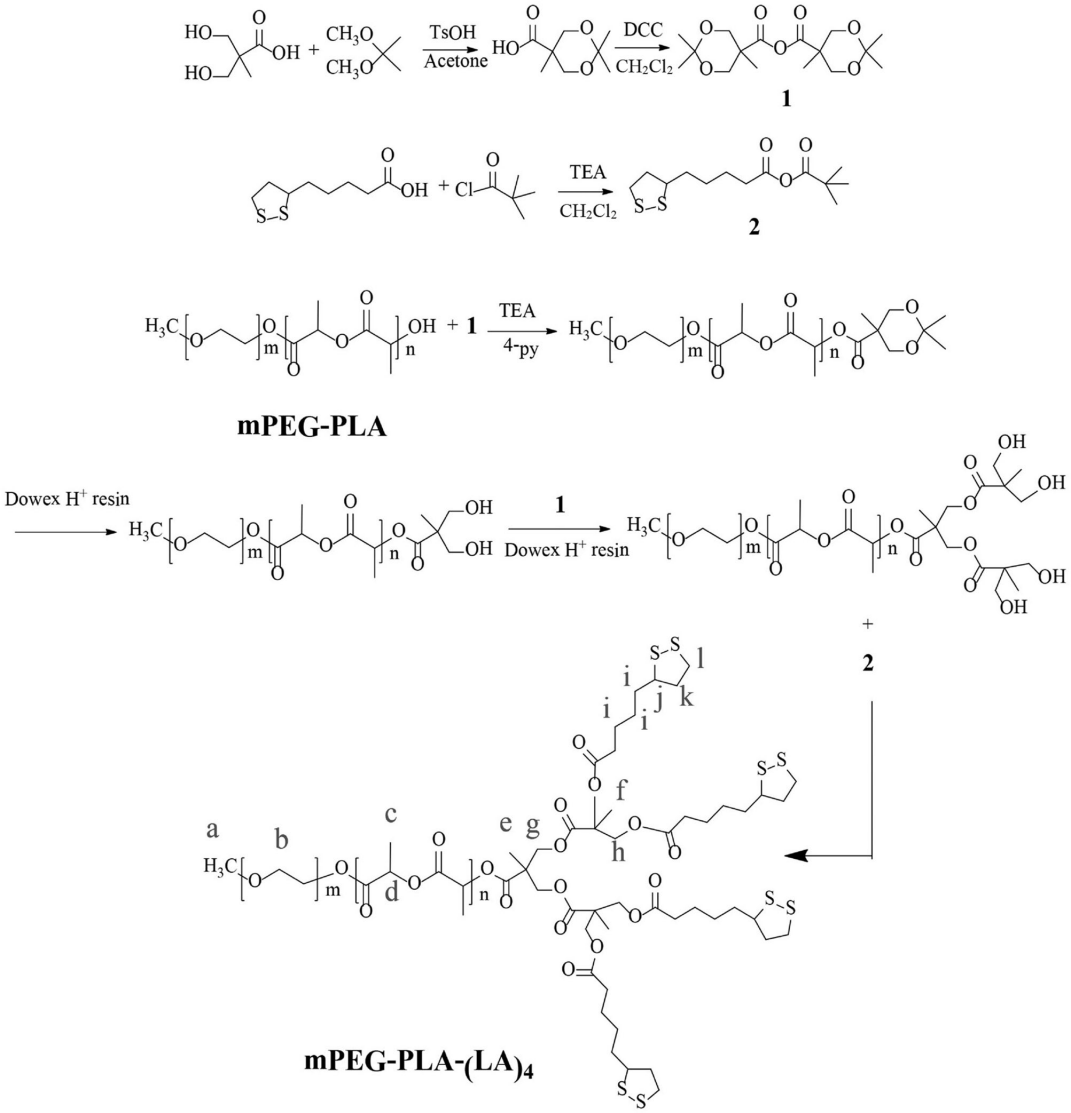
Synthetic route of mPEG-PLA-(LA)_4_.

**Figure 3.**
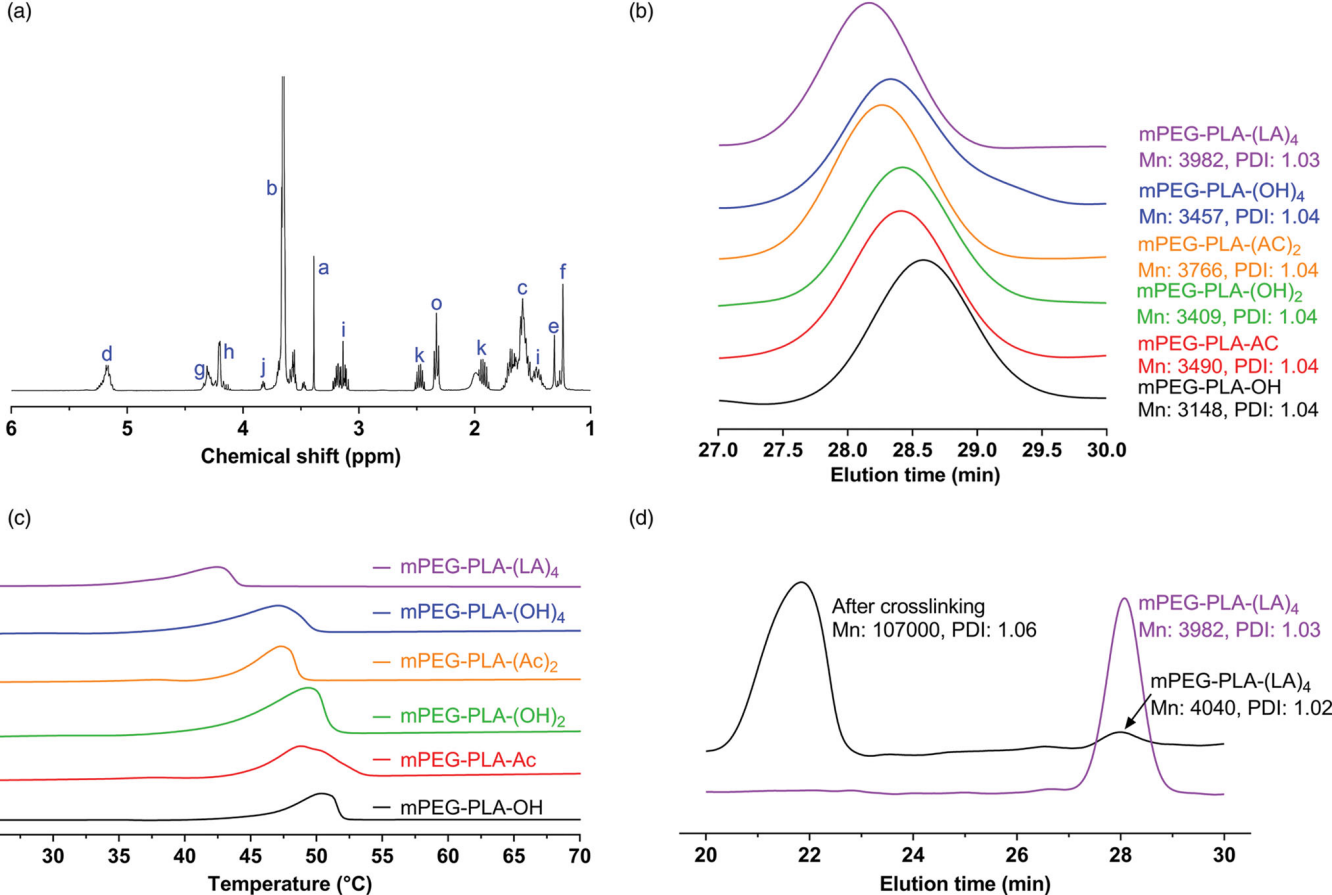
Characterization of mPEG-PLA-(LA)_4_. (a) ^1^H NMR spectrum of mPEG-PLA-(LA)_4_. (b) GPC curves of mPEG-PLA-OH, mPEG-PLA-Ac, mPEG-PLA-(OH)_2_, mPEG-PLA-(Ac)_2_, mPEG-PLA-(OH)_4_, and mPEG-PLA-(LA)_4_. (c) DSC thermograms of mPEG-PLA-OH, mPEG-PLA-Ac, mPEG-PLA-(OH)_2_, mPEG-PLA-(Ac)_2_, mPEG-PLA-(OH)_4_, and mPEG-PLA-(LA)_4_. (d) GPC curves of mPEG-PLA-(LA)_4_ before (blue) and after (black) crosslinking with 20% DTT.

The synthetic method was controlled precisely in every step, which led to a well-defined structure of the resulting dendritic lipoic acid functionalized block copolymer. The detailed synthetic route is illustrated in Figure 2. The first step was to synthesize hydroxyl-terminated mPEG-PLA-OH block copolymer via a ring-opening polymerization of d,l-lactide initiated by mPEG2000 and catalyzed by stannous octoate (Sn(Oct)_2_). After precipitation in cold ether, mPEG-PLA-OH was obtained as white powder up to a ∼90% yield. The molecular weight of the synthesized mPEG-PLA-OH was calculated from the ^1^H NMR spectrum (Figure 3(a)). The *M*_n_ of the mPEG block was 2000 g/mol and the molecular weight of the PLA block was 500 g/mol with a polydispersity index (PDI) of 1.04, as characterized by GPC analysis (Figure 3(b)).

The second coupling reaction of terminal hydroxyl groups in mPEG-PLA-OH with second generation acetonide-terminated polyester dendrons based on 2,2-bis(hydroxymethyl) propionic acid was achieved with a simple divergent growth approach (Gillies & Frechet, 2002). In the present study, 4-py was used as the condensing agent, which was found to be quite efficient and easy to be removed by simple re-crystallization from ethanol. The complete end-capping of terminal hydroxyl groups by polyester dendrons was confirmed by the ^1^H NMR spectrum (Figure 3(a)) and the GPC analysis (Figure 3(b)). The average end-capping ratio of mPEG-PLA-OH with polyester dendrons was determined by comparing the integration ratio between the signal *a*, which was assigned to the terminal methyl group in mPEG unit at 3.47 ppm, and the signal *h*, which was assigned to the methylene groups in the dendron unit with the theoretical value of 3:8 for 100% end-capping. In all cases, the ratios larger than 90% indicated total conversation of the end structure.

The third step was to synthesize the lipoic acid-terminated telodendrimer shown as mPEG-PLA-(LA)_4_ in Figure 2. Although the carboxyl groups in lipoic acid can also react with the hydroxyl end groups in mPEG-PLA-(OH)_4_ in the presence of acylating catalyst such as dicyclohexylcarbodiimide (DCC) (Gotsche et al., 1995), this direct coupling reaction was barely possible because of the low reactivity of the terminal hydroxyl groups in polymer chains. In the present study, an anhydride mixture of lipoic acid and pivaloyl chloride was used as the acylating agent to convert the terminal hydroxyl groups of mPEG-PLA-(OH)_4_ into the lipoic acid structure, which was found to be a powerful acylating reagent yielding complete conversion while it was not detrimental to the polymer backbone, as evidenced in the ^1^H NMR spectrum (Figure 3(a)) (Noda et al., 1990; Fan et al., 2005). After the coupling reaction, new peaks at 3.10–3.20, 2.44–2.49, 2.30–2.35, and 1.89–1.94 ppm, attributed to the lipoic acid, are shown in Figure 3(a), indicating the formation of the expected macromolecule. The end-capping efficiency was also calculated to be about 100% by comparing the integration ratio between the signal of the terminal methyl group in mPEG at 3.47 ppm and the signals of *i*, *k*, and *o* in the lipoyl unit. As described above, the terminal hydroxyl groups in mPEG-PLA-(OH)_4_ were completely capped by the mixed anhydride. The GPC profiles in Figure 3(b) also confirmed that the end-capping reaction of mPEG-PLA-(OH)_4_ resulted in a slight increase of about 600 g/mol in the polymer molecular weight, while the PDI remained constant. Altogether, our results clearly recapitulate the complete end-capping of the terminal hydroxyl groups in mPEG-PLA-(OH)_4_ with lipoic acid without changing the backbone of the block copolymer.

DSC thermograms revealed endothermic peaks for mPEG-PLA-OH, mPEG-PLA-Ac, mPEG-PLA-(OH)_2_, mPEG-PLA-(Ac)_2_, mPEG-PLA-(OH)_4_, and mPEG-PLA-(LA)_4_ (Figure 3(c)). All scans were run up to 100 °C and no thermal changes above 60 °C were observed for all the samples. All the end-functionalized polymers exhibited single exothermal peak below 51 °C, the melting point of mPEG-PLA-OH. The endothermic peaks for the block copolymers between 36 °C and 51 °C were probably due to the melting of mPEG region in the copolymers, indicating the formation of separated phases. The conjugation of amorphous dendritic structure with semi-crystalline mPEG-PLA-OH indeed suppressed the crystallinity of the block copolymer. The reduced melting temperatures of mPEG region in the block copolymers compared with pure mPEG indicated a lower degree of crystallinity in the copolymers. As the molecular weight of the terminal dendritic structure improved, the melting temperature of the copolymers decreased. It is possible that the dendritic structure interfered with the crystallization of the mPEG block resulting in an imperfect crystal. Deprotection of the terminal acetonide groups results in a decrease of the melting point (*T*_m_) while the molecular weight of the copolymers also changed (Figure 3(c)).

### 3.2. Micelle formation

**Figure 4.**
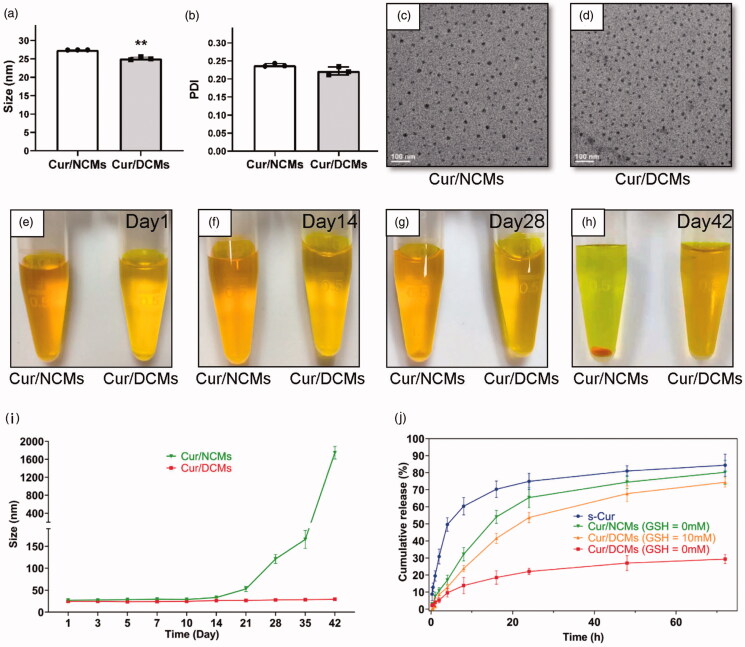
Characterization of Cur/NCMs and Cur/DCMs. (a) Sizes and (b) polydispersity index (PDI) of Cur/NCMs and Cur/DCMs. TEM images of (c) Cur/NCMs and (d) Cur/DCMs. Photographs of Cur/NCMs and Cur/DCMs kept at room temperature at (e) day 1, (f) day 14, (g) day 28, and (h) day 42. (i) Size evolution of Cur/NCMs and Cur/DCMs. (j) *In vitro* release profile of curcumin from s-Cur, Cur/NCMs, Cur/DCMs, and Cur/DCMs with 10 mM GSH. **p < 0.01

The first aim of this study was to develop blood stable and reduction sensitive reversibly disulfide crosslinked polymeric micelles for curcumin delivery, for which terminal dendronized mPEG-PLA and lipoic acid conjugates (mPEG-PLA-(LA)_4_) were designed and prepared. Cur loading in non-crosslinked micelles (Cur/NCMs) were prepared by a solid dispersion-thin film hydration method which can be easily scaled up (Gong et al., 2017). In this process, amphiphilic mPEG-PLA-(LA)_4_ self-assembled into spherical micelles (NCMs) with an average diameter of ∼25.4 nm and a PDI of 0.239, measured by DLS. mPEG-PLA-(LA)_4_ exhibited a low critical micelle concentration (CMC) of 12 mg/L, as determined by fluorescence measurements using pyrene as a probe (data not shown). Loading of curcumin into NCMs was performed at a curcumin concentration of 3 mg/mL and a theoretical drug loading content (DLC) of 5.0 wt%. The loading efficiency was nearly up to 100% and the final particle sizes fell in the range of ∼27.3 nm and a PDI of 0.237 prior to crosslinking. Cur/NCMs could be readily crosslinked using a catalytic amount (20 mol% relative to the lipoyl units) of DTT to initiate the ring opening polymerization of lipoyl rings to form a linear polydisulfide (Meng et al., 2009). After crosslinking, no significant change in curcumin loading capacity was observed, while the molecular weight of the polymer composing the micelles was significantly increased from 3982 g/mol to 107,000 g/mol with a polydispersity of 1.0–1.1 **(**Figure 3(d)). DLS measurements revealed that the size of Cur/DCMs decreased to ∼24.6 nm while PDI remained low of 0.225 (Figure 4(a,b)) after crosslinking. TEM images demonstrated that both Cur/NCMs and Cur/DCMs exhibited a spherical morphology and a size distribution close to that determined by DLS (Figure 4(c,d)).

### 3.3. *In vitro* stability

In order to investigate whether the intra-micellar disulfide crosslinking can enhance the stability of curcumin loaded micelles against severe micelle-disrupting conditions, the average diameters and PDI of Cur/NCMs and Cur/DCMs upon ethanol dilution and SDS condition were monitored by laser particle size analyzer. It is well known that SDS was able to efficiently solubilize the amphiphilic block copolymers at high concentrations, resulting in destabilization of the polymeric micelles (Li et al., 2011), and ethanol can also solubilize the mPEG-PLA-(LA)_4_ block copolymer. As shown in Fig. S1, Cur/DCMs exhibited superior colloidal stability against 50% ethanol and 50% SDS extensive dilution. After each Cur/DCM dispersion (3.0 mg/mL) was mixed with the same volume of SDS aqueous solution (2.5 mg/mL) for 10 min, the particle size was recorded. The PDI of the Cur/DCMs showed a slight increase, while the mean diameter was almost unchanged. The constant particle size of the Cur/DCMs under similar condition over time indicated that such crosslinked micelles remained intact. In contrast, abundant small and large aggregates appeared in the Cur/NCMs dispersion accompanying the significantly increased size distribution of the original micelle particles, indicating that NCMs were dissociated into unimers in SDS solution. The presence of ethanol resulted in large aggregates of NCMs, but barely influenced on Cur/DCMs. Cur/NCMs and Cur/DCMs were dispersed in PBS buffer solution and maintained at room temperature. At day 28, precipitation was observed in Cur/NCMs, the precipitation was more obvious at day 42, while no precipitation was observed in Cur/DCMs over the investigated time range (Figure 4(e–h)). Following the evolution of the particle size of Cur/NCMs and Cur/DCMs, the dimension of Cur/NCMs remained constant for at least 14 days, thereafter the particle size increases remarkably. The particle size of Cur/DCMs is stable for at least 6 weeks (Figure 4(i)). These results indicated that the core-crosslinked structure conferred excellent colloidal stability.

### 3.4. *In vitro* release profile

The drug release profiles of free curcumin (s-Cur), Cur/NCMs, and Cur/DCMs were measured using the dialysis method (Figure 4(j)). Curcumin released from s-Cur and Cur/NCMs were rapid. About 60% and 32% of curcumin were released from s-Cur and Cur/NCMs, respectively, within the first 8 h. At 24 h, 75% and 65% of curcumin in s-Cur and Cur/NCMs were released, whereas only 22% of curcumin was released from Cur/DCMs and the slow drug release was sustained for more than one week (data not shown), indicating that the disulfide crosslinked micelle (DCM) core significantly improved the stability of the micelles. Furthermore, as predicted, the release rate of Cur/DCMs was significantly accelerated as the GSH concentration was similar to the intracellular level (10 mM). This drug release behavior induced by 10 mM GSH can be exploited to achieve minimized premature drug release during circulation *in vivo*, but triggered release upon internalization of the micelles into cancer cells.

### 3.5. *In vitro* biocompatibility

**Figure 5.**
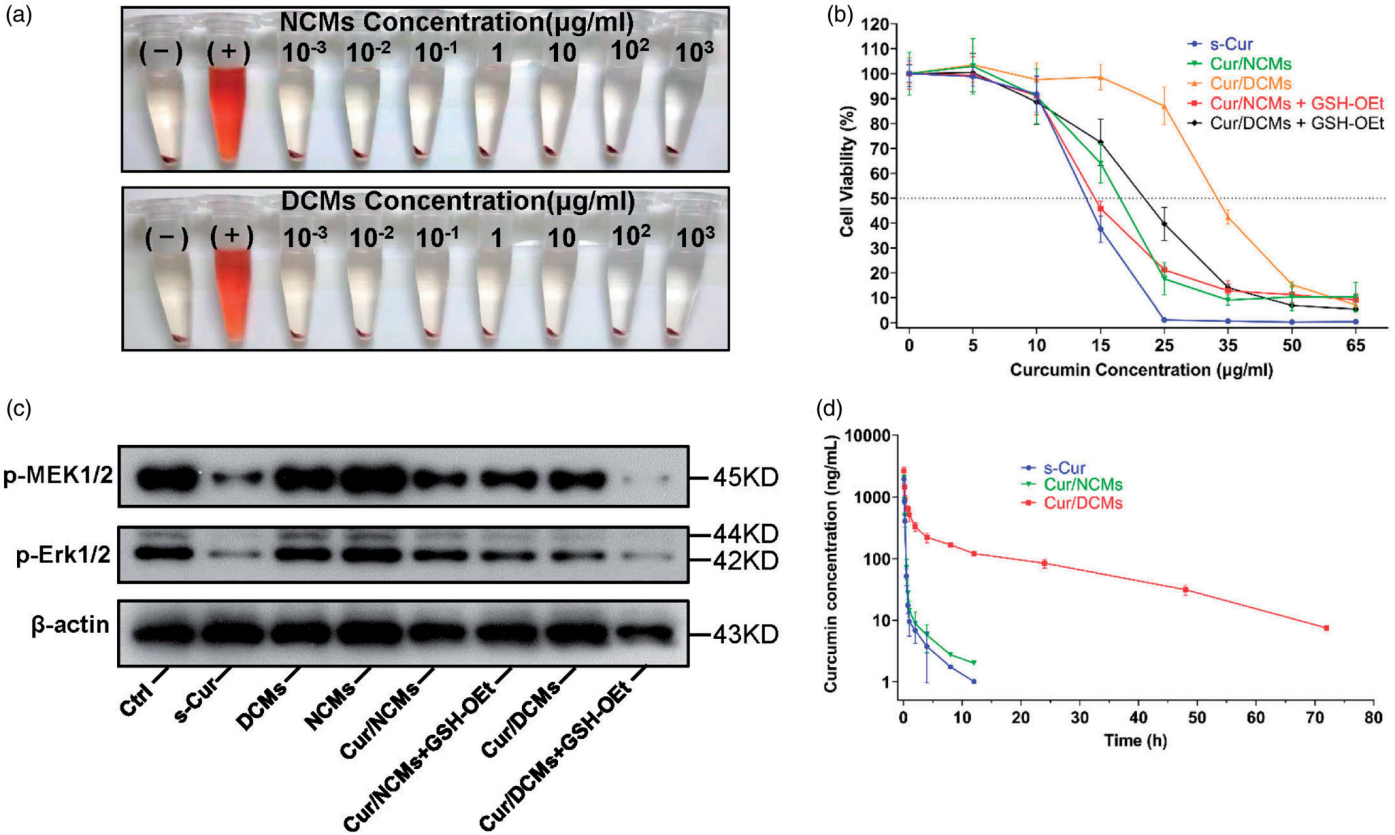
*In vitro* evaluation of the biocompatibility and anti-proliferation mechanism. (a) Photographs of RBCs after 3 h exposure to NCMs and DCMs at different concentrations (0.001–1000 μg/mL). The presence of red hemoglobin in the supernatant indicates RBCs with membrane damage. (+) in pure water and (–) in saline represent positive control and negative control, respectively. (b) Viability of MC-38 cells after incubation with s-Cur, Cur/NCMs, Cur/DCMs, Cur/NCMs + GSH-OEt, and Cur/DCMs + GSH-OEt for 72 h at various curcumin concentrations. The data are expressed as mean ± SD, *n* = 3. (c) The expression of p-MEK1/2 and p-Erk1/2 after treatment with s-Cur, DCMs, NCMs, Cur/NCMs, Cur/DCMs, Cur/NCMs + GSH-OEt, and Cur/DCMs + GSH-OEt for 10 h in MC-38 cells. (d) Plasma curcumin concentration–time curves after i.v. administration of s-Cur (dissolved in DMSO), Cur/NCMs, and Cur/DCMs in SD rats with a single dosage of 20 mg/kg.

*In vitro* hemolytic activity of NCMs and DCMs was evaluated with human red blood cells (RBCs). A universal method according to the literature for testing *in vitro* nanoparticle hemolysis was carried out (Dobrovoiskaia et al., 2008). With the increasing concentrations of both NCMs and DCMs up to 1000 μg/mL, the membrane of RBCs remained intact **(**Figure 5(a)), and hardly any free hemoglobin was detected in the supernatant (Fig. S2), indicating good biocompatibility with the blood.

The *in vitro* cytotoxicity of NCMs, DCMs, s-Cur, Cur/NCMs, and Cur/DCMs against MC-38 cancer cells was evaluated by a CCK8 assay. As shown in Fig. S3, blank NCMs and DCMs did not show detectable cytotoxicity even at a high polymer concentration of 1 mg/mL against tumor cells (MC-38) and the normal human colon mucosal epithelial cell line (NCM460) while s-Cur, Cur/NCMs, and Cur/DCMs exhibited dose-dependent cytotoxicity to MC-38 cells with the IC_50_ values of 13.7, 18.04, and 33.3 µg/mL, respectively (Figure 5(b)). Cur/DCMs showed much lower cytotoxicity than the solvent based formulation and the non-crosslinked formulation, most likely resulted from the much slower release rate of curcumin from the core crosslinked micelles. To estimate the GSH sensitivity of the core crosslinked micelles, the *in vitro* anticancer activity was also performed in MC-38 cells with an uplifted GSH level, which is well-known to break down the disulfide crosslinkage. GSH itself is not able to be effectively uptaken by cells owing to its anionic nature. Reports confirmed that GSH-OEt, a neutralized form of GSH, can penetrate cellular membranes and rapidly reach a high concentration of GSH through ethyl ester hydrolyzation in cytoplasm (Koo et al., 2008). In the present study, cells were pretreated with 10 mM GSH-OEt before incubation with different curcumin formulations to modulate the intracellular GSH concentration. As presented in Figure 5(b), cell viability treated with Cur/DCMs significantly decreased (*p*< 0.05) with 10 mM GSH-OEt pretreatment, while no significant difference of cell viability was observed for the Cur/NCMs treated group before and after GSH-OEt pretreatment. The IC_50_ values of Cur/NCMs + GSH-OEt and Cur/DCMs + GSH-OEt were 14.7 and 21.8 μg/mL, respectively. Considering the negligible cytotoxicity of blank NCMs and DCMs, the above cell growth inhibition was ascribed to the accelerated curcumin release from the core crosslinked micelles by the increased intracellular GSH concentration, which triggered the de-crosslinking of disulfide linkage in the micelle core.

Curcumin is known to play a role in anti-proliferation *via* modulating the MAPK signaling (Binion et al., 2008; Yallapu et al., 2014; Hsiao et al., 2020). We have further investigated whether the encapsulated curcumin function for mediating the anti-proliferation was through down-regulating p-MEK1/2 and p-ERK1/2 (Figure 5(c)), and the corresponding quantification is shown in Fig. S4. All groups containing curcumin (s-Cur, Cur/NCMs, Cur/DCMs, Cur/NCMs + GSH, Cur/DCMs + GSH) showed significantly low expression of the proliferative marker proteins. In particular, the triggered release of curcumin from DCMs exhibited the most intensified inhibition of cell proliferation.

### 3.6. Pharmacokinetics

Free curcumin in plasma was known to subject to rapid elimination and metabolism by the liver (Garcea et al., 2004); therefore, the *in vivo* stability is quite important in the carrier design for curcumin delivery. The present disulfide crosslinked formulation Cur/DCMs exhibited excellent stability that could protect the curcumin payload in the micelle core and therefore prolong the circulation time and improve the bioavailability after systemic administration.

A comparative pharmacokinetic study among free s-Cur, Cur/NCMs, and Cur/DCMs (curcumin 20 mg/kg) after i.v. administration in SD rats was performed. Plasma curcumin concentration–time curves are plotted in Figure 5(d). The main pharmacokinetic parameters of the three formulations were calculated using non-compartmental analysis, as listed in Table 1. The curcumin levels in SD rats at a single dose of 20 mg/kg from both s-Cur and Cur/NCMs declined rapidly and became lower than 100 ng/mL within 30 min and below the detection limit of 1 ng/mL after 12 h. Although curcumin can be encapsulated into the core of polymeric micelles to be completely dispersible in saline and intravenously injectable, most self-assembled micelles are reported to be dissociated by the blood components and lose their payload right after administration (Savic et al., 2006; Chen et al., 2008; Letchford & Burt, 2012). In the present study, no significant difference was observed in the pharmacokinetic behavior between s-Cur and Cur/NCMs, indicating that the bioavailability of curcumin was not improved by NCMs encapsulation. A distinct result was observed in the group treated with Cur/DCMs. The enhanced stability of curcumin by the core crosslinked micelles resulted in a significant increase in plasma curcumin concentration. Even after 48 h, the curcumin concentration in plasma from the group treated with Cur/DCMs was ∼30 ng/mL, which was equal to the serum concentration upon receiving a daily oral intake of 10 g (Cheng et al., 2001). For Cur/DCMs, the area under the time–concentration curve (AUC) was 7.55-fold larger, the half-life of elimination (*t*_1/2_) was 8.48-fold longer, the mean residence time (MRT) was 94.22-fold longer, and the maximum concentration in plasma was 1.49-fold higher than that of Cur/NCMs, while the total body clearance (CLz) was significantly decreased, indicating that elimination of curcumin was effectively decreased by core crosslinked micelle encapsulation. Taken together, these results demonstrated that Cur/DCMs markedly improved the stability of curcumin in the blood circulation, which obviously contributed to the improved bioavailability *in vivo*.

**Table 1.** Pharmacokinetic parameters after i.v. administration of s-Cur, Cur/NCMs, and Cur/DCMs in SD rats with a single dosage of 20 mg/kg.

Parameters	s-Cur	Cur/NCMs	Cur/DCMs
AUC_(0–_*_t_*_)_, µg L^–1^ h	1962.55	1932.62	14599.20
AUC_(0–∞)_, µg L^–1^ h	1965.17	1935.07	16139.57
MRT_(0–_*_t_*_)_, h	0.15	0.09	8.48
MRT_(0–∞)_, h	0.16	0.10	11.13
*t*_1/2_, h	1.39	1.00	8.48
*V*_z_, L kg^–1^	20.35	14.85	15.17
CL_z_, L h^–1^ kg^–1^	10.18	10.34	1.24
*C*_max_, ng L^–1^	7124.84	7223.06	10785.24

### 3.7. Antitumor efficacy

**Figure 6.**
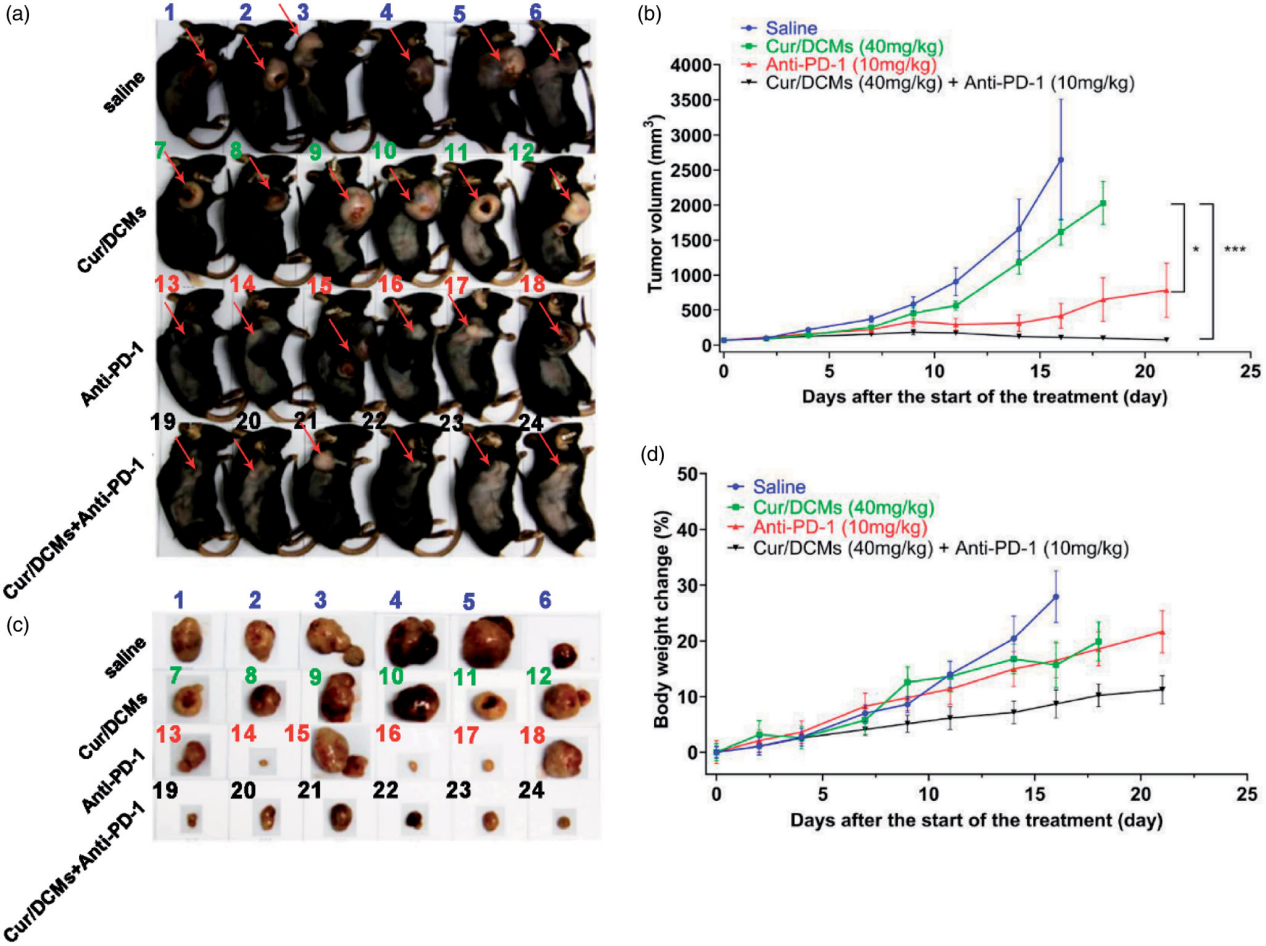
*In vivo* antitumor efficacy. (a) Photographs of MC-38 tumor bearing mice, euthanized at different endpoints. (b) Tumor growth curves in different groups after treatments. Each point represents the mean of tumor volume ± standard error, *n* = 6, *p< 0.05, ***p< 0.001. (c) Extracted tumors after mice were euthanized at different endpoints. (d) Body weight change (%) of mice after various treatments.

*In vivo* antitumor efficacy and systemic toxicity were evaluated on an MC-38 colon cancer xenograft model in C57BL/6 mice to examine the synergistic efficacy of Cur/DCMs with immunotherapy. First, as shown in Figure 6(a–c), the tumor volume in mice treated with saline grew rapidly, approximately 2600 mm^3^ on day 16. However, tumors in mice treated with Cur/DCMs were notably smaller than those in mice treated with saline. Cur/DCMs induced ∼40% of tumor growth retardant compared to the control saline group while anti-PD-1 greatly inhibited the tumor growth (*p*< 0.05). Second, enhanced tumor growth inhibition efficacy was observed by simultaneous administration of Cur/DCMs and anti-PD-1, the average tumor volume of which was only 9.6% of that in the only anti-PD-1 treated group. The results demonstrated that strong synergistic efficacy in treating cancer could be obtained through immunotherapeutic agent co-delivery with Cur/DCMs. More importantly, as compared to the anti-PD-1 and Cur/DCMs co-delivery group with tumor recurrence of 0%, the tumor recurrence of the group treated with single anti-PD-1 was 50% (three out of six mice). However, free s-Cur was found not to be able to significantly enhance the antitumor effects of anti-PD-1 in our previous research (data not shown), which indicated that free s-Cur had no synergistic anti-tumor efficacy on immunotherapeutic agent *in vivo* because of the instability of s-Cur during circulation in the bloodstream. In the present study, blood stable and reduction sensitive Cur/DCMs significantly improved free curcumin concentration in cancer cells through the EPR effect and selective burst release mechanism, which effectively reversed the immunosuppressive tumor microenvironment and improved the immunotherapeutic efficacy of anti-PD-1. The synergistic index is a key reference to evaluate the synergistic effect. Synergy refers to two or more components mixing together, and the effect is more remarkable than the sum of the effects deriving from individual components applied alone. We have tentatively calculated the synergistic index of the anti-PD-1 antibody combined with Cur/DCMs according to the reported method (Finney, 1952; Huang et al., 2019), and the value is 1.18. As the selected dosage was limited in our setting, the more accurate synergistic index resulting from additional experimental data will be carried out in our future experiments. Additionally, all the treatments were well-tolerated at the tested dosage and no apparent side effects, including body weight loss (Figure 6(d)), were observed in any group during the experiment.

## 4. Conclusions

We have designed and synthesized a telodendrimer (mPEG-PLA-(LA)_4_) capable of forming reversibly DCMs for *in vivo* curcumin delivery, and the *in vitro* cytotoxicity, pharmacokinetics, and antitumor efficacy with anti-PD-1 against MC-38 colon cancer have been investigated. The DCMs stably retained curcumin in the bloodstream and efficiently improved the systemic bioavailability, with a 7.55-fold larger of the AUC, 8.48-fold longer of the half-life of elimination (*t*_1/2_), 1.49-fold higher of the maximum concentration in plasma, and 94.22-fold longer of the MRT, as compared with the NCMs. Results in the antitumor setting further confirmed the synergistic anticancer efficacy of Cur/DCMs in combination with anti-PD-1 in treating MC-38 colon cancer. Therefore, our micellar formulation is expected to provide a feasible and efficacious way for delivering curcumin to reinforce the immunotherapy in treating cancers.

## Supplementary Material

Supplemental Material
