# Supplementary material for: Synergistic effect of the anti-PD-1 antibody with blood stable and reduction sensitive curcumin micelles on colon cancer
Source: Drug Deliv. 2021 May 11;28(1):930–42. doi: 10.1080/10717544.2021.1921077 (PMC8118404; doi:10.1080/10717544.2021.1921077)
Supplement: Supplemental Material [file IDRD_A_1921077_SM6077.docx]

**Synergistic effect of the anti-PD-1 antibody with blood stable and reduction sensitive curcumin micelles on colon cancer**

*Feirong Gong ^a,#^ ,* *Jian-Chao Ma ^b,c,#^, Jianguo Jia ^d,#^, Fa-Zhan Li ^b^, Jiao-Lan Wu ^b^, Shanfeng Wang ^e,*^, Xin Teng ^a,*^ and Zhong-Kai Cui ^b,c,^**

^a^Key Laboratory for Ultrafine Materials of Ministry of Education, School of Materials Science and Engineering, East China University of Science and Technology, Shanghai 200237, China

E-mail: tengxin@ecust.edu.cn

^b^Department of Cell Biology, School of Basic Medical Sciences, Southern Medical University, Guangdong 510515, China

E-mail: [zhongkaicui@smu.edu.cn](mailto:zhongkaicui@smu.edu.cn)

^c^ Guangdong Provincial Key Laboratory of Bone and Joint Degeneration Diseases, The Third Affiliated Hospital, Southern Medical University, Guangzhou, 510515, China

^d^ Department of Cardiology, Shanghai Institute of Cardiovascular Disease, Zhongshan Hospital, Fudan University, Shanghai 200032, China

^e^ School of Materials Science and Engineering, Sun Yat-sen University, Guangzhou 510275, China

E-mail: [wangshf25@mail.sysu.edu.cn](mailto:wangshf25@mail.sysu.edu.cn)

‡ These authors contributed equally to this work.


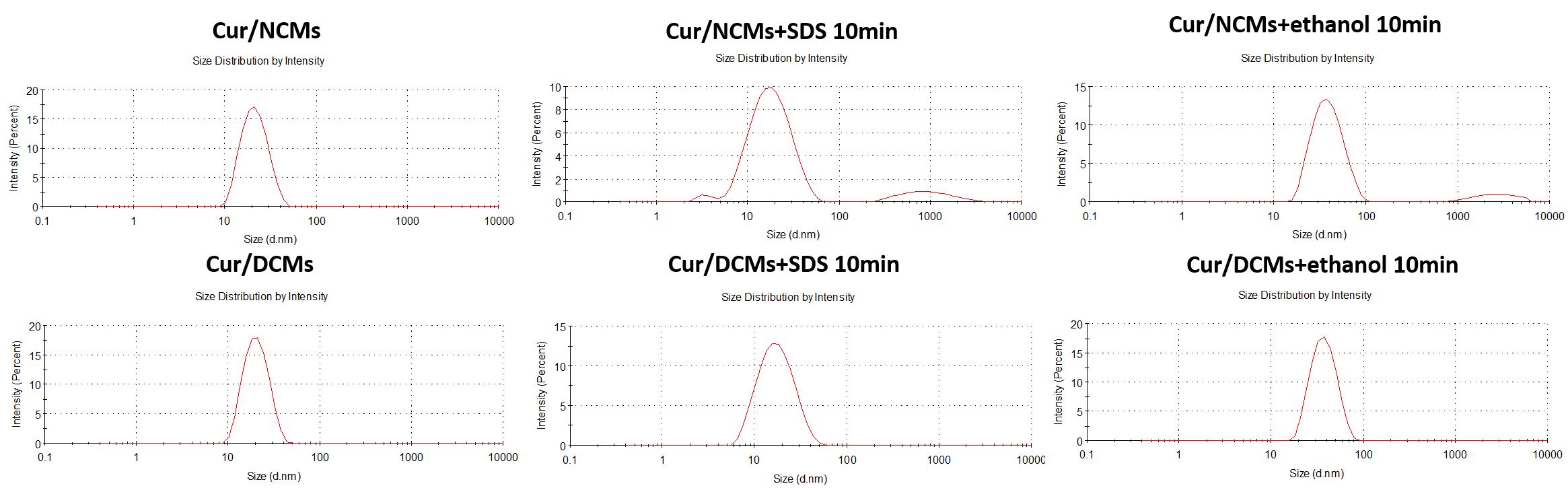


Figure S1. Size distribution of Cur/NCMs and Cur/DCMs in saline, 50% SDS, and 50% ethanol.


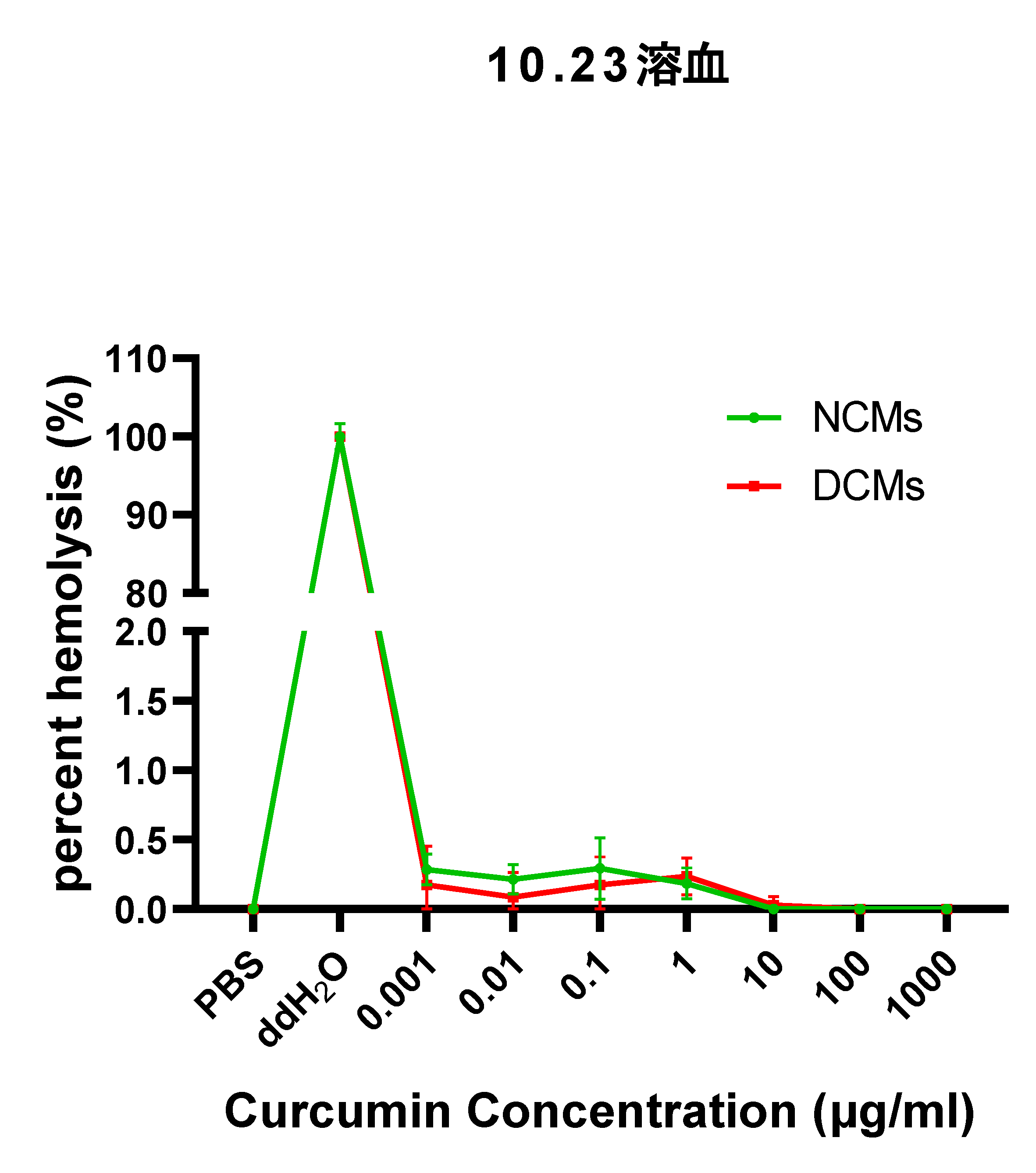


Figure S2. Percent hemolysis of RBCs incubated with different concentrations of NCMs and DCMs for 3 h at 37 ^o^C with agitation.


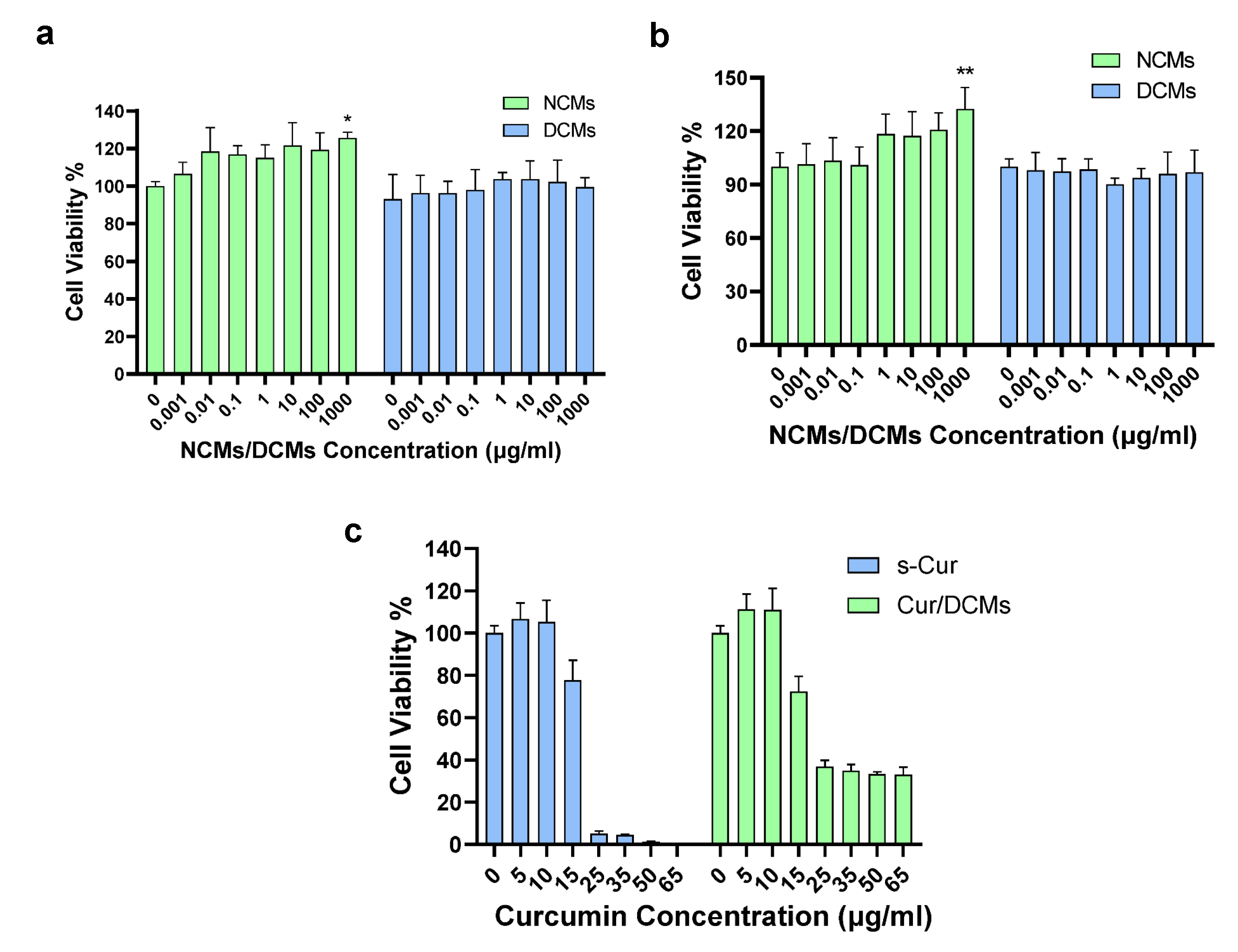


Figure S3. a) Viability of MC-38 cells after incubation with NCMs and DCMs for 72 h at various specified concentrations. b) Viability of NCM460 cells after incubation with NCMs and DCMs for 72 h at various specified concentrations. c) Viability of NCM460 cells after incubation with s-Cur and Cur/DCMs for 72 h at various specified curcumin concentrations. The data are expressed as mean ± SD, n = 3.


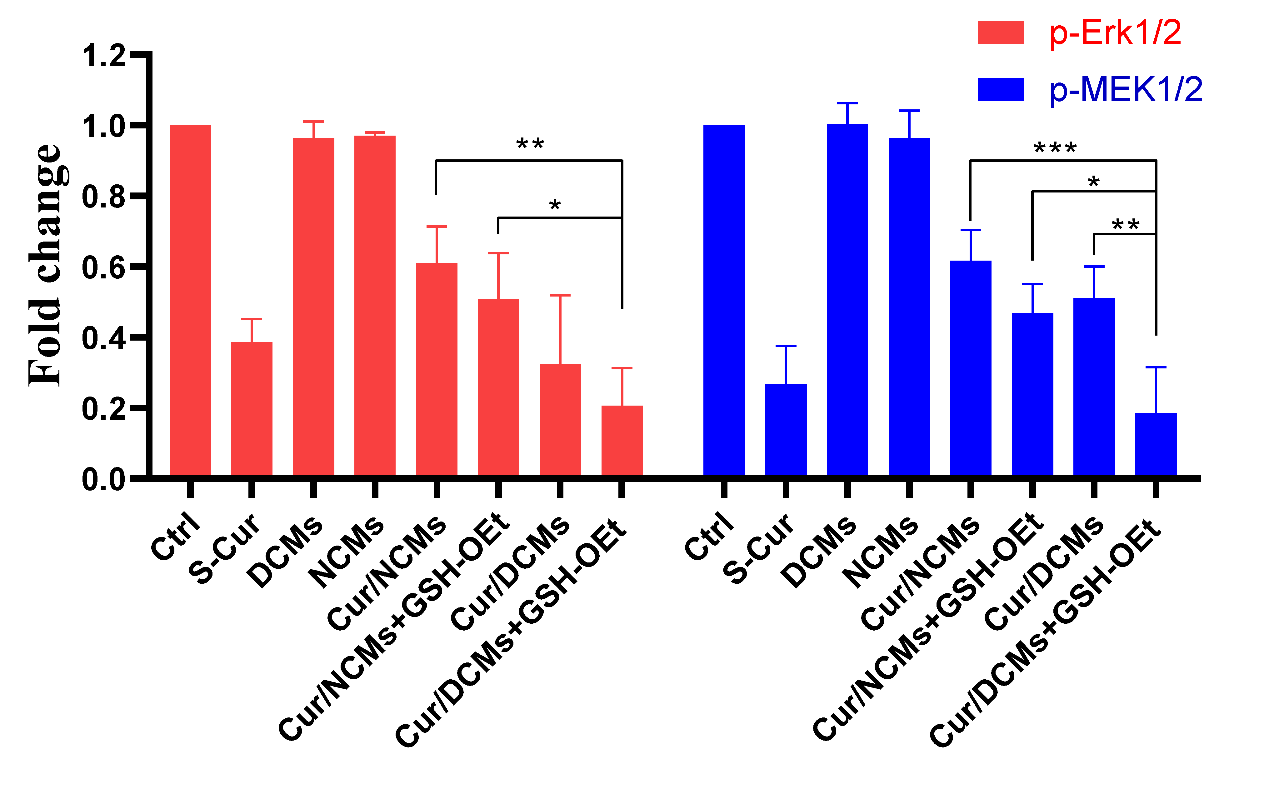


Figure S4. Quantification of the expression of p-MEK1/2 and p-Erk1/2 after treatment

with s-Cur, Cur/NCMs, Cur/DCMs, Cur/NCMs+GSH-OEt, and Cur/DCMs+GSH-OEt

for 10 h in MC-38 cells. The data are expressed as mean ± SD, n = 3, **p* < 0.05, ***p* <

0.01 and ****p* < 0.001.
